# Influence of augmentation of biochar during anaerobic co-digestion of *Chlorella vulgaris* and cellulose

**DOI:** 10.1016/j.biortech.2021.126086

**Published:** 2022-01

**Authors:** Jessica Quintana-Najera, A. John Blacker, Louise A. Fletcher, Andrew B. Ross

**Affiliations:** a School of Chemical and Process Engineering, University of Leeds, LS2 9JT Leeds, UK; b Institute of Process Research and Development, School of Chemistry, University of Leeds, LS2 9JT Leeds, UK; c School of Civil Engineering, University of Leeds, LS2 9JT Leeds, UK

**Keywords:** Anaerobic co-digestion, Biochar, Pyrolysis, Biomethane optimization, Microalgae

## Abstract

The anaerobic co-digestion (AcoD) of microalgae is a prospective option for generating biomethane from renewable sources. This study investigates the effects of inoculum-to-substrate ratio (ISR), C/N ratio and biochar (BC) load on the AcoD of *Chlorella vulgaris* and cellulose. An initial augmentation of BC at ISR 0.5–0.9 and C/N ratio 10–30 offered a pH buffering effect and resulted in biomethane yields of 233–241 mL CH_4_/g VS, corresponding to 1.8–4.6 times the controls. BC addition ameliorated significantly AcoD, supporting the digestate stability at less favourable conditions. The effect of the process variables was further studied with a 2^3^ factorial design and response optimisation. Under the design conditions, the variables had less influence over methane production. Higher ISRs and C/N ratios favoured AcoD, whereas increasing amounts of BC reduced biomethane yield but enhanced production rate. The factorial design highlighted the importance of BC-load on AcoD, establishing an optimum of 0.58 % (w/v).

## Highlights

• Biochar addition improved biomethane yield particularly at less favourable ISR 0.5–0.9.
• Biochar provided a buffering effect at lower ISR and C/N ratio.
• Factorial regression model provided optimal anaerobic co-digestion conditions.
• Biochar effect is highly dependent on the digestion conditions.

## 1 Introduction

Biomethane is a valuable option towards a sustainable and low-emissions future due to its compatibility with gas infrastructure. Currently, biomethane represents only 0.1% of natural gas demand, however, recent policy changes towards decarbonising transport are supporting its injection into natural gas grids. Many countries like Germany, Netherlands, United Kingdom, Brazil and the USA are supporting the introduction of biomethane into the transport sector. Since 2010, the installed biogas power generation capacity has been growing 4 % annually, however, this future development is highly dependent on feedstock availability (IEA, 2020).

Microalgae is an attractive feedstock for biofuel production due to their highly productive growth, and photosynthetic solar efficiency that doubles the terrestrial plants. Among the advantages of microalgae is the utilisation of land areas unsuitable for food production, utilise carbon dioxide emissions, resulting in lower land-use footprint and providing carbon–neutral biofuels. There are different types of microalgae, including the ‘weed’ green species *Chlorella vulgaris,* which is considered a rich biomass source, with a low content of toxic compounds, rapid growth rate and high protein content (Chronakis and Madsen, 2011). However, the demanding nutrient requirements and recalcitrant cell wall of *C. vulgaris* hinders its biodegradability (BD) (Ward et al., 2014). Increasing the BD of microalgae can be achieved by physical–chemical pre-treatments, however, this is often uneconomically or energetically unjustified.

Coupling microalgae cultivation with anaerobic digestion (AD) is suggested to overcome some of the inherent limitations (Ward et al., 2014). A circular process where nutrients from the anaerobic sludge are recovered for microalgae growth could improve economic and energy efficiency. Whereas the anaerobic co-digestion (AcoD) of microalgae with another carbon-rich substrate could improve the BD (Wang et al., 2013). AcoD of complex substrates offers several technological, ecological and economic advantages. A properly balanced co-digestion can provide synergistic effects, improve the process stability, methane yield, kinetic parameters, and in consequence the economic viability of biomethane (Li et al., 2017).

AcoD of an N-rich and a C-rich substrate at an adequate C/N ratio can supply the N requirements for microorganisms and alleviate pH changes due to acid products. The co-digestion of *C. vulgaris* with a reference substrate with known degradability, in this case cellulose, allows the effect of biochar augmentation on C/N ratios to be investigated and allows optimum C/N ratio to be controlled. Reports of improved methane yields of *C. vulgaris* in co-digestion with C-rich biomass have been attributed to adequate C/N ratios (Zhang et al., 2019). Adequate C/N ratios are often found at 15–30, although the optimum value is highly dependent on the feedstock (Yao et al., 2017). For instance, the optimal C/N ratio varied for crops 20 (Piątek et al., 2016), corn stover 25 (Yao et al., 2017), algae 25–30 (Bohutskyi et al., 2018), and microalgae 20–25 (Yen and Brune, 2007).

Other approaches could complement AcoD, such as implementing optimal inoculum to substrate ratio (ISR) and AD amendment by adding adsorbent carbon materials. The inoculation influences the initial activity and performance of the digester. Hence, ISR is an essential operating condition that needs to be evaluated for optimising digestion (De la Rubia et al., 2018). The implementation of optimum ISR helps maintain the digester stability, avoid the accumulation of volatile fatty acids (VFAs), and reduce the necessity of nutrient media supplementation while obtaining better methane yields (Okoro-Shekwaga et al., 2020).

The addition of biochar (BC) in AD is reported to enhance methane production rate and yields (Quintana-Najera et al., 2021), mitigate ammonium inhibition (Mumme et al., 2014), promote methanogenic metabolism, reduce the lag phase (Shanmugam et al., 2018), and improve the syntrophic oxidation of VFAs (Paritosh and Vivekanand, 2019). The positive effect of BC has been attributed to its advantageous physicochemical properties. A developed porosity provides a large surface area (SA) for the interaction and immobilisation of the cells. While the surface oxygenated functional groups (OFGs) are reported to serve as anchoring and interaction sites for biomolecules that facilitate direct interspecies electron transfer (DIET) interactions between microorganisms (Zhao et al., 2015). Nonetheless, not all BCs have the desired properties or positive effects on AD. Thus, the possibility to tailor the BC properties to fulfil desired characteristics by controlling the pyrolysis conditions strengths its applications and versatility (Lee et al., 2017).

Although many studies have reported AcoD for enhancing methane yields, the simultaneous addition of BC could provide another promising approach. Therefore, the current study was undertaken to establish the potential of BC for enhancing methane generation during the AcoD of *C. vulgaris* and cellulose and to identify the optimal digestion conditions. The first aim was to investigate the effect of BC at different C/N ratios and ISR on methane yields and kinetic parameters. The second aim was to use a factorial design 2^3^ for identifying the optimum BC load and ISR during the AcoD of *C. vulgaris* and cellulose at different C/N ratios.

## 2 Materials and methods

### 2.1 Inoculum, substrate and biochar

Anaerobic sludge collected from the mesophilic wastewater treatment plant Esholt in Bradford, United Kingdom was collected and stored at 4 °C. Before use, the inoculum was homogenised by passing it through a mesh (1 mm). The total solids (TS) and volatile solids (VS) were quantified gravimetrically (APHA, 2005). The substrates used were the microalgae *C. vulgaris* and cellulose. Autotrophic *C. vulgaris* was produced and dried in China and cracked in a ball mill at the University of Leeds. The composition of *C. vulgaris* was analysed as: i) biochemical (protein 40.5 %, lipids 15.6 %, and carbohydrates 36 %); ii) proximate (volatile matter 77.1 %, fixed carbon 14.3 % and ash 8.6 %); iii) ultimate (C 54.6, H 8.1, N 9.3, O 19.5 %). Proximate analysis was determined using a thermogravimetric analyser (TGA) Mettler Toledo (TGA/DSC 1). For elemental analysis, an automatic CHNS Thermo Instruments Flash (EA 1112 Series) was employed with values expressed as a percentage of total dry weight, with total oxygen (O) determined by difference. Cellulose was selected as the C-rich substrate because as a model substrate it facilitated the C/N ratio calculation, and is regarded as a reference for other agricultural feedstocks since it often represents their primary component. Oak wood BC was produced at 450 °C at commercial pyrolysis operated by Proininso (Spain), its physicochemical properties were characterised elsewhere (Quintana-Najera et al., 2021).

### 2.2 Biochemical methane potential

Biochemical methane potential (BMP) measurement was performed with the Automatic Methane Potential Test System (AMPTS II) (Bioprocess Control, Sweden) and calculated as expressed in **Eq.** (1). The AcoD experiments were performed in 500 mL reactors with a working space of 400 mL. After filling the reactors with the desired conditions, they were flushed with nitrogen for establishing anaerobic conditions. The reactors were incubated at 37 °C for 30 days and automatically stirred for 60 s every 10 min.

(1) $$\mathrm{BMP}=\frac{\mathrm{Volume}\;{\mathrm{CH}}_{4}\;from\;sample\;\left(mL\right)-Volume\;{\mathrm{CH}}_{4\;}from\;blank\;\left(mL\right)}{g\;VS\;ofsubstrate\;fed\;in\;digester}$$

### 2.3 Anaerobic co-digestion of microalgae and cellulose

The conditions for the first AcoD experiments consisted of inoculum 5 g VS/L, BC load 0 and 3 % (w/v), cellulose 5 g VS/L and variable amounts of *C. vulgaris* for achieving C/N ratios of 10, 20 and 30, respectively. The necessary amount of *C. vulgaris* was calculated based on the chemical composition of both substrates. The increasing amount of *C. vulgaris* for obtaining C/N ratios of 10, 20 and 30 reduced the ISRs to 0.5, 0.8 and 0.9, respectively. Controls consisting of inoculum and substrate at each C/N ratio and ISR, without BC, alongside a blank consisting only of inoculum to account for residual methane emissions were performed in parallel. The treatments with BC at each C/N ratio were performed by triplicate, whereas the controls and blank were performed by duplicate. The C/N ratios ranged between optimum values (20–30) and sub-optimal (10). The objective of this was to establish the potential of the BC in ameliorating critical processing conditions during the AcoD of microalgae and cellulose.

### 2.4 Theoretical biochemical methane potential

The theoretical BMP (BMP_Th_) of a substrate can be estimated from its chemical composition according to the Boyls equation (**Eq.** (2)). This equation assumes a substrate breakdown efficiency of 100 % and considers only the products CH_4_ and CO_2_. The ash content was subtracted from the calculation and only the biodegradable fraction was considered, hence the BMP_th_ is expressed as mL CH_4_/g VS. **Eq.** (2) is calculated at STP conditions (273 K, 1 atm), where c, h, o and n represent the molar fractions of C, H, O and N, respectively (Aragón-Briceño et al., 2020, Buswell and Mueller, 1952). The values for *C. vulgaris* (BMP_th,a_) and cellulose (BMP_th,b_) were further used to establish the BMP_th_ of their combination according to **Eq.** (3). C_a_ and C_b_ are their corresponding mass fraction used at the different C/N ratios.

(2) $${\mathrm{BMP}}_{\mathrm{th}}=\frac{22400*(\frac{c}{2}+\frac{h}{8}-\frac{o}{4}-\frac{3n}{8})}{12c+h+16o+14n}$$

(3) $${\mathrm{BMP}}_{\mathrm{th}}=\left({\mathrm{BMP}}_{\mathrm{th},a}*C_{a}\right)+\left({\mathrm{BMP}}_{\mathrm{th},b}*C_{b}\right)$$

### 2.5 Anaerobic biodegradability

The anaerobic BD of methane for each treatment considered the combination of substrates at a given C/N ratio. BD was calculated from the final experimental BMP and the BMP_Th_ according to **Eq.** (4) (Aragón-Briceño et al., 2020).

(4) $$BD\left(\%\right)=\frac{BMP}{{\mathrm{BMP}}_{\mathrm{Th}}}*100$$

### 2.6 Kinetic analysis

The experimental BMP values were fitted to the modified Gompertz model (**Eq.** (5)) according to Zwietering et al. (1990).

(5) $$\mathrm{BMP}\left(t\right)={\mathrm{BMP}}_{\max}∙exp\left\{-exp\left[\frac{\mu_{m}∙e}{{\mathrm{BMP}}_{\max}}\left(\lambda -t\right)+1\right]\right\}$$

where; BMP(t) = Cumulative methane yield (mL CH_4_/g VS) at time t (day), BMP_max_ = Maximum methane yield (mL CH_4_/g VS), µ_m_ = Methane production rate (mL CH_4_/g VS·day), λ = Lag phase (days), e = exp(1).

### 2.7 Factorial design 2^3^

To determine the optimum processing conditions for the AcoD of *C. vulgaris* and cellulose, a full factorial 2^3^ experimental design was performed. The study comprised three independent factors at two levels, C/N ratio (7 and 25), ISR (1 and 2) and BC load (0 and 3 %) with 3 replicates and 3 centre points (C/N 16, ISR 1.5 and BC load 1.5 %). The inoculum was fixed at 10 g VS/L, whereas the amount of substrate added ranged from 5 to 10 g VS/L for achieving the corresponding ISR. The amount of *C. vulgaris* and cellulose added for each C/N ratio and ISR were calculated based on their chemical composition. For achieving the C/N ratio of 7, 16 and 25, the ratio of *C. vulgaris* to cellulose were 0.8:0.2, 0.3:0.7 and 0.2:0.8, respectively. A factorial regression model was used for analysing biomethane production (Montgomery, 2013). The desirability (D) function was used for optimising the AcoD conditions. The main objective of this experiment was to investigate and optimise the effect of BC load and ISR under variable C/N ratios.

### 2.8 Analytical methods

The amount of VFAs accumulated at the end of the AcoD was measured by gas chromatography. An Agilent 7890A gas chromatograph, a DB-FFAP column (30 m × 0.32 mm, film thickness of 0.5 µm) and a flame ionisation detector (FID) at 200 °C with nitrogen as make-up gas was used. An autosampler injected 10 µL of the sample at a 5:1 split ratio. The inlet port operated at 150 °C with helium at 10 mL/min as a carrier gas. The column oven started at 60 °C for 4 min, then increased to 140 °C with a ramp of 10 °C/min, and finally raised to 200 °C with a ramp of 40 °C/min and held for 5 min. A volatile acid standard mix (Supelco) and alcohols made from high purity single reagents were used as comparative standards. Data was acquired with ChemStation software. Ammonia can be found in the digester in the form of free or non-ionised ammonia (NH_3_) and ionised ammonium (NH_4_^+^). Ammonia was detected by a spectrophotometer (DR 3900, HACH) using the LCK305 ammonium cuvette test 1.0–12.0 mg/L NH_4_-N/NH_3_-N test based on the indophenol blue method and according to ISO 7150–1 and DIN 38,406 E5-1 water quality standards. Total ammonia nitrogen (TAN) is expressed as the sum of NH_4_-N and NH_3_-N. The solid fraction decanted at the end of the digestion was oven-dried and evaluated by CHNOS elemental analysis.

### 2.9 Statistical analysis

The SPSS Statistics 26 software was used for most statistical analyses and to calculate the Gompertz kinetic parameters. While Minitab 27 software was used for creating the factorial design and analysing the response variables, models and optimisation. Comparison of the effect of the factors C/N ratio, ISR and BC load over the data was performed by analysis of variance (ANOVA) and linear regression at a confidence level of p < 0.05.

## 3 Results and discussion

### 3.1 Exploratory anaerobic co-digestion experiments

#### 3.1.1 Experimental biochemical methane potential

**Fig. 1:**
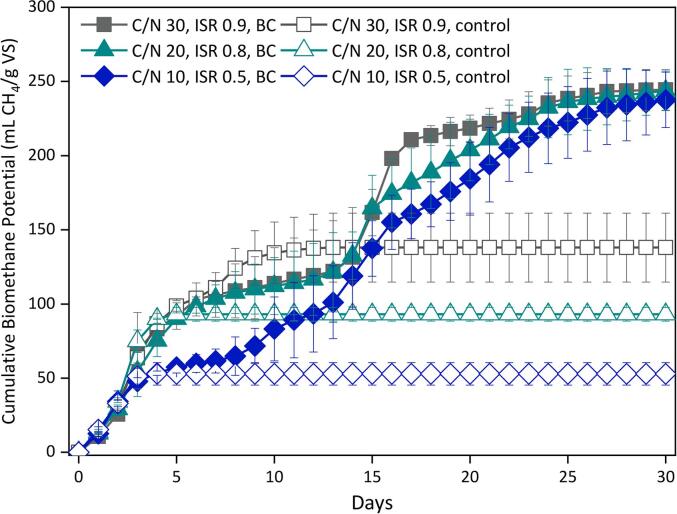
Effect of biochar augmentation on the biochemical methane potential for the exploratory anaerobic co-digestion of cellulose and *Chlorella vulgaris* at C/N ratios 10–30 and ISRs 0.5–0.9.

Fig. 1 shows the cumulative BMP with and without the addition of BC during the AcoD of cellulose and *C. vulgaris* at 10–30C/N ratios and ISR 0.5–0.9. All systems started generating methane since day one, although the controls rapidly reached maximum production and steady-state. Conversely, the BC systems showed an initial plateau, followed by second exponential and stationary phases.

For the controls, reducing the C/N ratio and ISR resulted in lower BMP yields due to less favourable conditions. Nonetheless, the C/N ratio and ISR showed no statistically significant effect over the final BMP_Exp_ yield (p > 0.05). The detrimental impact of reducing the C/N below the optimal could be attributed to ammonia accumulation and toxicity (Bohutskyi et al., 2018). Also to the low ISR range employed, since ISR below 0.8 is reported to facilitate the proliferation of acidogens and acetogens and inhibition of methanogens (Moset et al., 2015). The BMP was similar for the BC systems at C/N ratios of 10, 20 and 30, achieving final yields of 233, 239 and 241 mL CH_4_/g VS, respectively. These values corresponded to an enhance of 4.6, 2.6 and 1.8 times their control, respectively. The dramatic improvement of BMP yields due to the BC addition suggested that BC could have ameliorated the co-digestion of microalgae and cellulose, particularly at less favourable conditions.

#### 3.1.2 Kinetic modelling

Table 1 shows the kinetic parameters obtained by fitting the experimental BMP to the modified Gompertz model. BMP_max_ at the C/N ratios of 10, 20 and 30 was 82, 66 and 51 % higher for the systems augmented with BC in comparison to their control, respectively. The controls quickly reached the steady-state, which was reflected in their higher µ_m_ in comparison to the BC systems. Both variables BMP_max_ and µ_m_ had a statistically significant difference with the BC addition (p < 0.05). However, neither the C/N ratio nor ISR showed significance over these kinetic parameters (p > 0.05). Increasing the C/N ratio and ISR reduced the period of the lag phase, although not significantly (p > 0.05). BC addition was the factor with the highest influence over the AD kinetic parameters, suggesting that BC positively influenced AcoD performance.

**Table 1:** Biomethane experimental yield and kinetic parameters obtained with the modified Gompertz model for anaerobic co-digestion of cellulose and *Chlorella vulgaris* with the addition of oak wood biochar.

**Systems**	**Experimental data**				**Modified Gompertz model**			
	**BMP_Th_(mL CH_4_/g VS)**	**BMP_Exp_(mL CH_4_/g VS)**	**BD(%)**		**BMP_max_(mL CH_4_/g VS)**	**µ_m_(mL CH_4_/g VS·day)**	**λ(days)**	**R^2^**
C/N 10, ISR 0.5, BC	506.5	232.7	46.0		278.6	9.5	1.0	0.987
C/N 10, ISR 0.5, control	506.5	50.8	10.0		50.9	23.6	0.4	0.993
C/N 20, ISR 0.8, BC	459.6	239.1	52.0		270.2	10.0	0.0	0.972
C/N 20, ISR 0.8, control	459.6	91.2	19.8		91.4	39.5	1.0	0.993
C/N 30, ISR 0.9, BC	444.4	241.2	54.3		275.5	12.4	0.0	0.975
C/N 30, ISR 0.9, control	444.4	136.2	30.7		136.2	22.7	0.5	0.994

BMP_Exp_ maximum experimental methane yield, BD biodegradability; BMP_max_ maximum methane yield; µ_m_ methane production yield; λ duration of lag phase; BC load of 3 % (w/v),

#### 3.1.3 Biodegradability

The BMP_Th_ for *C. vulgaris* (607 mL CH_4_/g VS) is considerably higher than cellulose (415 mL CH_4_/g VS) due to the high content of protein and lipids of the former (Bohutskyi et al., 2018). Hence, the differences in BMP_Th_ were a consequence of the amount of microalgae added for achieving each C/N ratio. The BD with the addition of BC and non-BC controls at the three C/N ratios ranged at 46–54 % and 10–31 %, respectively (Table 1). The achieved BD values were far from the theoretical maximum which is partially attributed to the limiting BD of microalgae due to its thick and recalcitrant cell wall. Even though *C. vulgaris* was physically pre-treated by ball-milled cracking. The BD could only improve up to a certain extent since it has been stated that a fraction of undigested microalgae usually remains intact throughout the AD process (Ward et al., 2014). Furthermore, the BDs were generally lower in comparison to previous BMP tests where the same concentration of cellulose was added as mono-substrate (BD 64–70 %) (Quintana-Najera et al., 2021). Hence, an inhibitory effect originated principally by *C. vulgaris* and low ISR could have hindered the BD and BMP values, less drastically for the systems supplemented with BC than the controls and directly correlated to the reduction of C/N ratio.

#### 3.1.4 Volatile fatty acids and pH

**Fig. 2:**
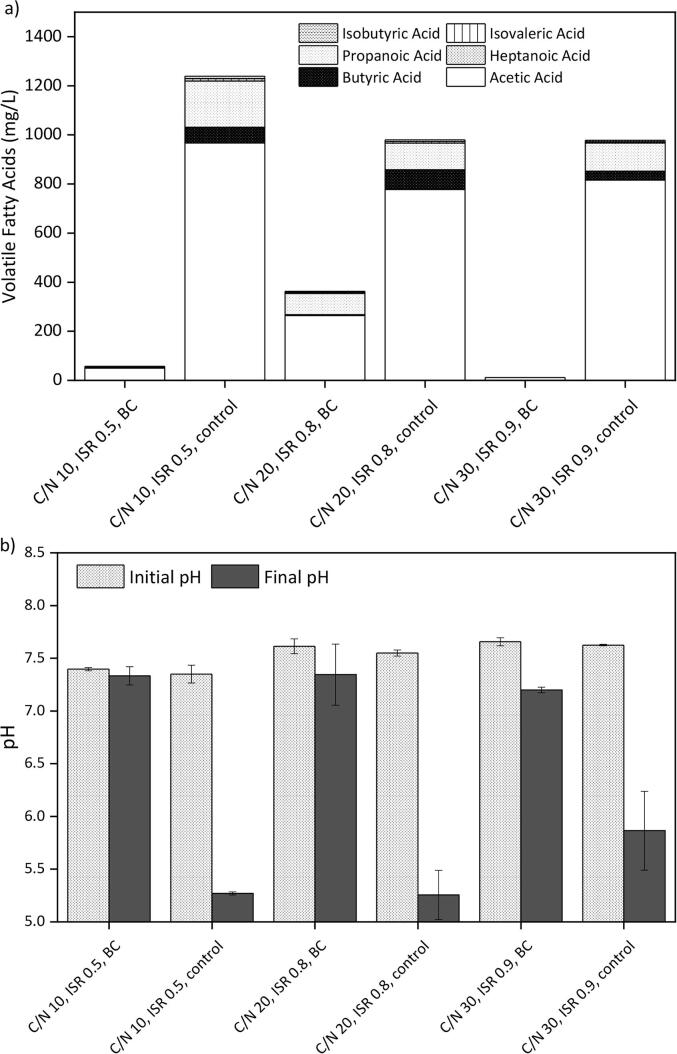
Effect of biochar augmentation on the anaerobic co-digestion of cellulose and *Chlorella vulgaris* at C/N ratios 10–30 and ISRs 0.5–0.9. a) Accumulated volatile fatty acids; b) pH.

Fig. 2a shows the VFAs accumulated at the end of the AcoD experiments. The systems that produced lower amounts of BMP also resulted in higher VFAs accumulation. The controls (C/N 20, ISR 0.8) and (C/N 30, ISR 0.9) showed a similar accumulation of VFAs, whereas the control (C/N 10, ISR 0.5) reached 1239 mg total VFAs/L. On the other hand, the BC systems at C/N ratios of 10 and 30 exhibited negligible VFAs. Although the system (C/N 20, ISR 0.8, BC) accumulated 363 mL total VFAs/L. Nevertheless, no system reached toxic levels since acetic inhibition on methanogens is reported to take place at concentrations above 1619 mg/mL (Xiao et al., 2013).

Fig. 2b shows the pH measured at the beginning and the end of the AcoD process. All systems started with a similar pH (7.4–7.7), which by the end of the fermentation suffered negligible variations on the reactors supplemented with BC. The controls, on the other hand, suffered a drastic pH reduction (pH 5.3–5) that intensified at lower C/N ratios and ISR. The changes in pH agree with the BMP_Exp_ values, as those systems whose pH suffered more variation also produced less BMP. The latter suggests that the oak wood BC here used could have provided a buffering effect given its alkaline nature (pH 9.9). Similarly, there are reports of BC having a positive buffering role in AD (Paritosh and Vivekanand, 2019, Wang et al., 2018). A digester with adequate alkalinity would stabilise the AD process from variations of VFAs and pH. However, if the alkalinity is insufficient, the digester would undergo acidosis and the methane production would cease (Sterling et al., 2001).

#### 3.1.5 Biochar in anaerobic co-digestion

BMPs and BD were hindered with the increasing addition of *C. vulgaris* (C/N reduction) and in consequence the increase of N-content. This behaviour was drastically observed for the non-BC controls, whereas it was considerably milder for the BC systems. The better BMP performance in the presence of BC could be attributed to the beneficial properties of BC, such as alkalinity, conductivity, SA and possible role as a facilitator of syntrophic metabolism via DIET interactions (Klüpfel et al., 2014, Quintana-Najera et al., 2021). The characterisation of the oak wood BC produced at 450 °C, used for this work was detailed in a previous publication. The BC showed a developed SA of 221 m^2^/g and pore volume (PV) of 0.09 m^3^/g and large availability of OFGs (Quintana-Najera et al., 2021). The SA and PV could provide a suitable environment for interaction and/or adsorption of molecules between the different microorganisms. While the OFGs, often dominated by quinone/hydroquinone functionalities, could have been responsible for redox buffering capacity and facilitators of DIET interactions (Klüpfel et al., 2014). Therefore, this experiment demonstrated that BC addition could fulfil the necessity of an alkaline source, avoid drastic changes in pH and maintain the stability of the AD process under the conditions here studied. However, it is desirable to establish the best blend ratios for the substrates and inoculum for achieving positive synergisms, nutrient balance, avoid inhibition, and optimise methane productivity (Mata-Alvarez et al., 2011). Hence, the following section evaluates the optimum conditions for the AcoD of microalgae and cellulose to obtain a better BMP performance.

### 3.2 Factorial design

#### 3.2.1 Experimental biochemical methane potential

**Fig. 3:**
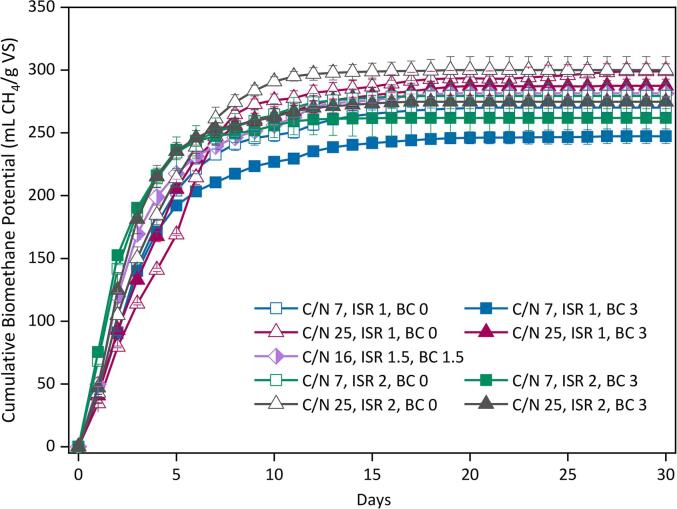
Biochemical methane potential for the anaerobic co-digestion of cellulose and *C. vulgaris* for the factorial design 2^3^ conditions.

Fig. 3 shows the average BMP produced by each condition of the factorial design for the AcoD of *C. vulgaris* and cellulose. All systems started producing methane since day one exhibiting a quick exponential phase while reaching the steady-state by the 10th day of digestion. The final BMP yields differed by up to 17 % among the conditions since they were found in a range of 247–299 mL CH_4_/g VS (Table 2). The highest BMP yield was obtained by the system (C/N 25, ISR 1.0, BC load 0 %), while the lowest yield was obtained at (C/N 7, ISR 1.0, BC load 3.0 %). BMP yields were enhanced by increasing the C/N ratios due to more favourable conditions. Increasing the BC load appeared to reduce BMP yields, whereas ISR showed no consistent trend.

**Table 2:** Average biomethane experimental yield, biodegradability, nitrogen fate and kinetic data for the anaerobic co-digestion of *Chlorella vulgaris* and cellulose obtained for the experimental design.

**Independent variables**			**Experimental**				**Modified Gompertz**		
**C/N**	**ISR**	**BC load**	**BMP_Exp_(mL CH_4_/g VS)**	**BD(%)**	**TAN(mg NH_3_-N/L)**	**N (%)**	**BMP_Max_(mL CH_4_/g VS)**	**µ_m_(mL CH_4_/g VS·d)**	**λ(d)**
7	1	0	270.1	47.3	20.8	4.3	265.9	45.3	0.0
7	1	3	247.3	43.3	30.3	4.0	242.2	42.8	0.0
7	2	0	277.6	48.6	17.2	4.0	274.9	59.0	0.0
7	2	3	259.0	45.3	24.3	4.1	258.6	70.2	0.0
16	1.5	1.5	275.0	57.7	19.5	3.1	274.0	49.5	0.0
25	1	0	298.9	65.9	17.2	3.9	294.2	41.8	0.4
25	1	3	287.5	63.4	22.2	3.0	283.7	44.4	0.1
25	2	0	295.7	65.2	15.5	3.3	298.5	49.7	0.1
25	2	3	272.5	60.1	17.1	3.0	270.2	66.2	0.2

BMP_Exp_ maximum experimental methane yield; BD biodegradability; TAN total ammonia nitrogen measured on the supernatant; N content measured in the decanted solid by ultimate CHNS analysis; BMP_max_ maximum methane yield; µ_m_ methane production yield; λ duration of lag phase.

The final BMP yields assuming additive behaviour obtained from the mono-AD of *C. vulgaris* and cellulose with and without BC addition is compared to the actual BMP data observed during co-digestion and is shown in the supplementary data. The additive BMP for the combination of cellulose and *C. vulgaris* at each C/N ratio was calculated based on the fraction of each substrate and the final BMP obtained from their mono digestion (supplementary material). This allowed a comparison of any synergy during co-digestion with or without the addition of BC.

A synergistic increase in BMP was observed during co-digestion of the two substrates in comparison to predicted additive behaviour based on mono-digestion. This improvement was observed at most conditions of the DoE, particularly at lower ISR and C/N ratios (supplementary material). Increasing the ISR improved the mono digestion of the two substrates, in agreement with other reports (Zhang et al., 2019). However, this was also accompanied by an increase in BMP during co-digestion.

The amount of synergistic increase in BMP during co-digestion was reduced when adding 3 wt% BC, however when optimum BC loading was added (0.6 wt%), the synergistic increase was higher than co-digestion without BC. This illustrates the importance of BC loading as well as the benefits of co-digestion of an additional carbon source for the digestion of *C. vulgaris*.

#### 3.2.2 Kinetic modelling

Table 2 shows the kinetic parameters obtained with the modified Gompertz model. The values of BMP_max_ were gradually improved as the C/N ratio increased and the BC load reduced. The period of lag phase was almost negligent for all systems, while µ_m_ showed the greatest variation. The highest µ_m_ of 70.2 and 66.2 mL CH_4_/g VS·d, were obtained at the conditions (C/N 7, ISR 2.0, BC 3.0) and (C/N 25, ISR 2.0, BC 3.0), respectively. The rest of the systems exhibited values of 40–55 mL CH_4_/g VS·d, corresponding to 16–39 % lower µ_m_ than the best performers. The effect of the C/N ratio showed no evident trend over µ_m_ while increasing both ISR and BC load resulted in the most significant enhancement.

#### 3.2.3 Biodegradability

Table 2 shows the BMP_Th_ for the AcoD of *C. vulgaris* and cellulose at each condition. As previously stated, variable BMP_Th_ is obtained from the mixture of the substrates at the different C/N ratios. The BMP_Th_ at the C/N ratios 7, 16 and 25 corresponded to 571, 477 and 454 mL CH_4_/g VS, respectively. The differences in BMP_Th_ in addition to the BMP_Exp_ dramatically affected the BD values, which ranged at 43–66 %. Even though the BMP_Th_ increased when lowering the C/N ratio, the actual BMP and BD values were reduced due to the complexity, recalcitrance, and difficulty to degrade *C. vulgaris* (Bohutskyi et al., 2018). Hence, an inhibitory effect originated principally by reducing ISR, increasing the content of *C. vulgaris*, and in consequence, reducing the C/N ratio could have hindered the BD values.

#### 3.2.4 Fate of inorganic nitrogen

To study the fate of organic nitrogen at the end of AcoD of microalgae and cellulose, both solid and liquid phases were separated and analysed (Table 2). The TAN on the liquid phase was measured by spectrophotometry, while the N-content on the decanted solid was quantified by elemental CHNS analysis. TAN values were considerably low for all experimental conditions (17–30 mg/L), slightly higher with BC addition and at a lower C/N ratio. This behaviour contrasts the reported by Lu et al. (2019) for the AcoD of *Chlorella* sp. with septic tank sludge. They obtained a final TAN of approximately 200–2300 mg/L, although even these levels showed no inhibitory effect over methanogenic activity. The N content of the remaining solids comprised by the digestate and BC was slightly higher at a lower C/N ratio and without BC addition, although the general values were similar to the N content of the inoculum (3.7 %). This behaviour, in addition to the BMP yields and BD, suggests adequate digestion of the microalgae, without ammonia inhibition even at the lowest C/N ratios.

#### 3.2.5 Volatile fatty acids and pH

**Fig. 4:**
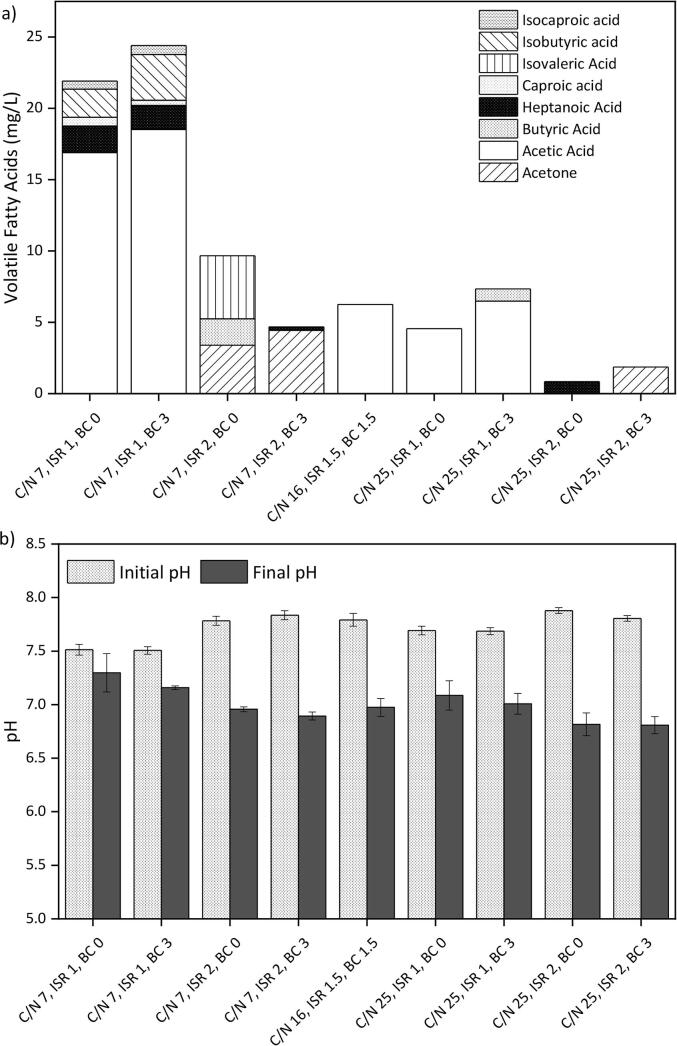
Anaerobic co-digestion of cellulose and *C. vulgaris* for the factorial design 2^3^ conditions. a) Accumulated volatile fatty acids accumulated; b) pH.

Fig. 4a exhibit the VFAs accumulated at the end of the AcoD experimental design. The less favourable conditions in terms of lower ISR and C/N resulted in a greater VFA accumulation. Nevertheless, such low concentrations could be considered negligible in all cases. Fig. 4b shows the pH at the beginning and end of the AcoD. No pH adjustment was performed to evaluate the effect of the processing conditions C/N ratio, ISR and BC load. The initial pH (7.5–7.9) was similar for all systems, although higher than the optimal levels for the AD process (pH 6.7–7.4). By the end of the digestion, the systems exhibited different pH variations with final pH of 6.8–7.3, within the optimal range. Increasing ISR and C/N ratio reduced the final pH, with a statistically significant effect (p < 0.05). This effect is contrary to the expected since higher ISR and balanced C/N ratios are associated with pH buffering. Even though BC is an alkaline additive and it previously proved to offer a buffering effect on AcoD, under these conditions its addition had no effect on the pH (p > 0.05).

#### 3.2.6 Regression model fitting

The parameters BMP_Exp_, BMP_max_ and µ_m_ were selected as response variables for the analysis of the factorial design. The regression models obtained were statistically significant with p < 0.05 and F-value > F-critical at the 0.05 alpha level (Supplementary data). The centre points included in the 2^3^-design protected against curvature from second-order effects, validating the fitting of the first-order regression model (p < 0.05). **Eq. 6, 7** and **8** show the factorial regression models for BMP_Exp_, BMP_max_ and µ_m_. These regressions exhibited R^2^ of 0.73, 0.85 and 0.84, respectively. Hence, only 15–27 % of the variability cannot be explained by the models. The adjusted R^2^ 0.69–0.80 values were fitted to the actual size of the model and the number of factors, whereas the prediction R^2^ 0.58–0.67 indicates the variability that the model would explain during the prediction of new data. In brief, the significance and fitting of the quadratic models to the experimental data were satisfactory.

BMP_Exp_ = 275.95 + 12.58*CN + 0.13*ISR − 9.53*BC – 4.69*CN*ISR (6)

BMP_max_ = 273.58 + 13.13*CN + 2.00*ISR − 9.86*BC – 4.32*CN*ISR (7)

µ_m_ = 52.11 + 8.83*ISR + 3.48*BC + 3.45*ISR*BC (8)

From the analysis of variance, each factor and interaction of factors offered a specific coefficient and p-value (at 95 % confidence) as listed in Table 3. The significant specific coefficients (p < 0.05) for the factors and interactions are part of the regression models. Even though the factor ISR was non-significant for BMP_Exp_ and BMP_max_ (**Eq. 6** and **7**), the coefficients were kept based on the hierarchy principle. This principle promotes internal consistency by indicating that if a model contains a high order term (CN*ISR), it must contain all the lower order terms (CN and ISR) (Montgomery, 2013). The factors C/N ratio and BC load influenced both BMP_Exp_ and BMP_max_, with no significant effect from ISR. The C/N ratio did not affect the response variable µ_m_, which was influenced exclusively by ISR and BC load and their interaction.

**Table 3:** Statistical evaluation of the factors and interactions comprising the factorial regression models.

**Coefficient probability**						
	**BMP_Exp_**		**BMP_max_**		**µ_m_**	
Term	Coefficient	p-value	Coefficient	p-value	Coefficient	p-value
Constant	275.95	0.000	273.58	0.000	52.11	0.000
CN	12.58	0.000	13.13	0.000	−1.91	0.080
ISR	0.13	0.951	2.00	0.246	8.83	0.000
BC	−9.53	0.000	−9.86	0.000	3.48	0.003
CN*ISR	−4.69	0.047	−4.32	0.018	−1.42	0.185
CN*BC	1.69	0.705	0.15	0.927	1.28	0.231
ISR*BC	−0.95	0.671	−1.30	0.447	3.45	0.003
CN*ISR*BC	−1.98	0.380	−3.15	0.075	0.02	0.984

BMP_Exp_ maximum experimental methane yield; BMP_max_ maximum methane yield, µ_m_ methane production yield.

#### 3.2.7 Influence of main factors and interactions

In this experiment, the factor ISR showed no statistically significant differences in BMP_Exp_ and BMP_max_ (Table 3). Moset et al. (2015) reported that regardless of the substrate used, the BMP enhanced as the ISR increased over a range of 1.5–2.5, although not significantly. Similarly, De la Rubia et al. (2018) studied the influence of ISR from different inoculum sources on the AD of the process water obtained from the hydrothermal carbonisation of dewatered sewage sludge. When using sewage sludge inoculum, they observed that the ISR had no significant difference over BMP. These reports agree with the negligent impact of ISR on BMP observed in this experiment. Regardless of the nature and complexity of the substrate used, the BMP was not affected by ISR if an appropriate range is selected (ISR 1.0–2.0).

ISR enhanced the response variable µ_m_ showing a statistically significant effect. The initial inoculum concentration is reported to influence the rate of substrate hydrolysis. Hence, higher ISR often results in faster fermentation and consequence enhanced production rate (Raposo et al., 2011). Similarly, Raposo et al., (2009) studied the impact of ISR 0.8–3.0 on the AD of sunflower oil cake. They observed a maximum production rate at ISR 2.0. However, unlike the linear trend observed in this study, they obtained higher µ_m_ at ISR 2.0 > 1.0 > 3.0 > 0.8 > 1.5 > 0.5.

The impact of microalgae and cellulose addition to varying the C/N ratio showed a significant difference over BMP_Exp_ and BMP_max_ but not over µ_m._ The range of C/N ratio selected for this experiment started at an optimal ratio of 25 and moved downward to less favourable conditions. Hence, higher BMP yields were obtained according to the following C/N ratio order 25 > 16 > 7. Similarly, Bohutskyi et al. (2018) observed a synergistic effect by co-digesting algae and cellulose. They reported the highest BMP yields and production rate at C/N ratios of 21 and 34 than lower ratios or even the mono-digestion of each substrate. Therefore, increasing the C/N ratio enhanced BMP yields but did not influence the production rate.

BC load had a statistically significant effect on all variables (p < 0.05). For BMP yield, the coefficient of BC load had a negative value, which indicates that increasing the BC load would result in lower BMP. This response contrasts to the observed in the previous section, where BC drastically enhanced BMP yields at ISR 0.5–0.9 and C/N ratio 10–30. On the other hand, increasing BC load led to higher µ_m_ which partially agrees with previous experiments with the addition of this same oak wood BC. The addition of BC at a load of 3 % during the AD of cellulose slightly enhanced BMP yields (7 %), whereas it doubled µ_m_ (Quintana-Najera et al., 2021). Reports of BC addition during the AD of microalgae demonstrated the importance of BC load. Deng et al. (2020) studied the AD of *Laminaria digitata* and *Saccharina latissima* at variable BC loads. For *L. digitata*, a BC addition of 0.06 and 0.125 % enhanced BMP yields and µ_m_, whereas higher BC loads of 0.5 and 1.0 % reduced both parameters. Conversely, for the AD of *S. latissima* BC loads < 0.5 % had no significant influence, whereas BC load of 0.5 and 1.0 enhanced both BMP and µ_m_. The latter suggests that BC load influenced BMP yields and production rate, but the effect level was subjected to the substrate employed. Therefore, it is necessary to establish the optimum BC load for achieving the highest BMP yield and productivity for each potential substrate.

#### 3.2.8 Optimisation of biomethane

**Fig. 5:**
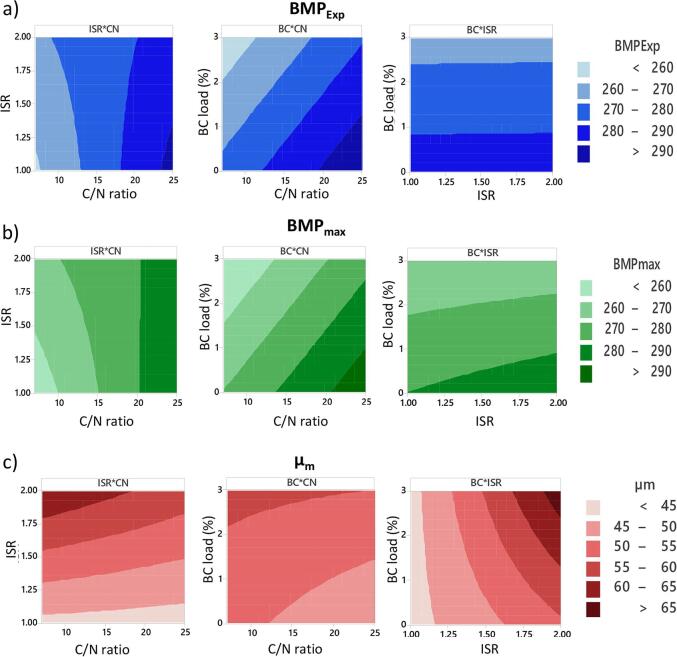
Contour plots for interaction effects and optimised area obtained by response surface regression. a) Experimental methane yield (BMP_Exp_); b) maximum methane yield (BMP_max_); c) methane production rate (µ_m_).

Graphical interpretation of the responses facilitates the examination of factors and interactions in regression models. Contour plots with a combination of the three factors C/N, ISR and BC were used for visualising the optimum areas for each response variable (Fig. 5). As expected, the contour plots for BMP_Exp_ and BMP_max_ are similar. The stretching of the axis indicates that maximum values can be obtained at C/N 22–25 and BC 0–1.5 regardless of the ISR. The contour plots for µ_m_ differed since maximum values were obtained at ISR of 1.7–2.0 and BC load of 1–3, regardless of the C/N ratio. The interaction plots for BMP showed that the C/N ratio played a major role (Fig. 5a and b). This factor also interacted strongly with the rest, indicating a predominant influence. In the case of µ_m_, ISR exhibited the major effect, while its interaction with BC load was significant on the response in agreement to the regression models (Fig. 5c).

The response variables BMP_EXp_, BMP_max_ and µ_m_ were analysed by the D function for the factorial regression optimisation. The objective was to improve these parameters to achieve maximum suitability. Composite D assessed how the combined variables satisfy the response. Hence, the optimum conditions obtained were C/N 25, ISR 2.0 and BC load 0.58 % with the highest possible BMP of 294 mL CH_4_/g VS, and µ_m_ of 57 mL CH_4_/g VS·d. A D-value of 0.62 indicated that all responses were predicted to be within the desired limits. A further AcoD experiment under these optimal conditions is shown as supplementary data. The obtained final BMP (312 mL CH_4_/g VS·d) was even higher than the predicted, supporting the relevance of the model.

Response optimisation for obtaining maximum biomethane allowed the prediction and evaluation at other C/N ratios. For instance, as the C/N ratio is reduced to 16 and 7, the optimum ISR must be maintained at 2, while BC load decreased at 0.34 and 0 %, respectively. These experimental conditions will result in BMP yields of 284 and 275 mL CH_4_/g VS and µm of 55.6 and 54.0 mL CH_4_/g VS·d, respectively. Regression model and response optimisation probed to be useful when working with variable C/N ratios due to variability in substrate composition and availability.

## 4 Conclusions

This study demonstrated the importance of the C/N ratio, ISR and BC load during the anaerobic co-digestion of *C. vulgaris* and cellulose. ISR 0.5–0.9 exhibited low BMP yields, which were considerably improved by BC addition that also provided a pH buffering effect. Regression models and optimisation analysis demonstrated that as the C/N ratio is reduced, the BC load should also be reduced to achieve better performance. Hence, the beneficial effect of BC addition was more visible at higher C/N ratios, suggesting that the BC effect is highly dependent on the digestion conditions.

## CRediT authorship contribution statement

**Jessica Quintana-Najera:** Conceptualization, Investigation, Data curation, Formal analysis, Methodology, Project administration, Resources, Software, Validation, Visualization, Writing – original draft, Writing – review & editing. **A. John Blacker:** Conceptualization, Investigation, Project administration, Resources, Supervision. **Louise A. Fletcher:** Conceptualization, Investigation, Supervision. **Andrew B. Ross:** Conceptualization, Funding acquisition, Investigation, Project administration, Resources, Supervision, Validation, Visualization, Writing – review & editing.

## Declaration of Competing Interest

The authors declare that they have no known competing financial interests or personal relationships that could have influenced the work reported in this paper.
