# Supplementary material for: Influence of augmentation of biochar during anaerobic co-digestion of Chlorella vulgaris and cellulose
Source: Bioresour Technol. 2022 Jan;343:126086. doi: 10.1016/j.biortech.2021.126086 (PMC8633764; doi:10.1016/j.biortech.2021.126086)
Supplement: Supplementary data 1 [file mmc1.docx]

**Influence of augmentation of biochar during anaerobic co-digestion of *Chlorella vulgaris* and cellulose**

Jessica Quintana-Najera^a^, A. John Blacker^a,b^, Louise A. Fletcher^c^, and Andrew B. Ross^a,^^[[1]](#footnote-1)^*

^a^School of Chemical and Process Engineering, University of Leeds, LS2 9JT Leeds, UK

^b^Institute of Process Research and Development, School of Chemistry, University of Leeds, LS2 9JT Leeds, UK

^c^School of Civil Engineering, University of Leeds, LS2 9JT Leeds, UK

**Supplementary data**

**Table S1**

Analysis of variance for the factorial regression models.

| **Analysis of Variance (ANOVA)** | | | |
| --- | --- | --- | --- |
| **Variable** | **BMP_Exp_** | **BMP_max_** | **µ_m_** |
| R^2^ | 0.73 | 0.85 | 0.84 |
| Adjusted R^2^ | 0.69 | 0.80 | 0.79 |
| Prediction R^2^ | 0.58 | 0.67 | 0.66 |
| F | 15.24 | 15.51 | 14.64 |
| F_critical_ | 2.51 | 2.51 | 2.51 |
| Model p-value | 0.00 | 0.00 | 0.00 |
| Lack of fit p-value | 0.882 | 0.932 | 0.367 |

**Fig. S1.** Comparison of the final methane obtained from the experimental design conditions, a further AcoD under optimal conditions and the calculated from the mixture of the substrates based on their mono-digestion values.

The BMP normalised (BMP_Norm_) was calculated based on the BMP of the mono-digestion of each substrate, at ISRs 1 and 2, and the amount added for achieving each C/N ratio. The BMP at optimal conditions (BMP_opt_) confirmed the optimised conditions for the regression model.

**Fig S2.** Biomethane production for anaerobic co-digestion of cellulose and *Chlorella vulgaris* under optimal conditions C/N 25, ISR 2 and char load 0.58 % (w/v).

OW-BC450 corresponded to oak wood biochar produced at 450 °C. The control consisted of inoculum and substrate at C/25 and ISR 2

1. *Corresponding author

   E-mail address: [a.b.ross@leeds.ac.uk](mailto:a.b.ross@leeds.ac.uk) (A. Ross) [↑](#footnote-ref-1)
